# Synthesis, Structural, and Cytotoxic Properties of New Water-Soluble Copper(II) Complexes Based on 2,9-Dimethyl-1,10-Phenanthroline and Their One Derivative Containing 1,3,5-Triaza-7-Phosphaadamantane-7-Oxide

**DOI:** 10.3390/molecules25030741

**Published:** 2020-02-08

**Authors:** Ewelina I. Śliwa, Urszula Śliwińska-Hill, Barbara Bażanów, Miłosz Siczek, Julia Kłak, Piotr Smoleński

**Affiliations:** 1 Faculty of Chemistry, University of Wrocław, ul. F. Joliot-Curie 14, 50-383 Wrocław, Poland; ewelina.sliwa@chem.uni.wroc.pl (E.I.Ś.); milosz.siczek@chem.uni.wroc.pl (M.S.); julia.klak@chem.uni.wroc.pl (J.K.); 2 Department of Analytical Chemistry, Faculty of Pharmacy, Wroclaw Medical University, Borowska 211 A, 50-566 Wrocław, Poland; urszula.sliwinska-hill@umed.wroc.pl; 3 Department of Pathology, Wrocław University of Environmental and Life Sciences, ul. Norwida 31, 50-375 Wrocław, Poland; barbara.bazanow@upwr.edu.pl

**Keywords:** copper(II), discrete complexes, 2,9-dimethyl-1,10-phenanthroline, 1,3,5-triaza-7-phospaadamantane-7-oxide, antitumor activity, cytotoxic activity

## Abstract

A series of water-soluble copper(II) complexes based on 2,9-dimethyl-1,10-phenanthroline (dmphen) and mixed-ligands, containing PTA=O (1,3,5-triaza-7-phosphaadamantane-7-oxide) have been synthesized and fully characterized. Two types of complexes have been obtained, monocationic [Cu(NO_3_)(O-PTA=O)(dmphen)][PF_6_] (**1**), [Cu(Cl)(dmphen)_2_][PF_6_] (**2**), and neutral [Cu(NO_3_)_2_(dmphen)] (**3**). The solid-state structures of all complexes have been determined by single-crystal X-ray diffraction. Magnetic studies for the complex **1**–**3** indicated a very weak antiferromagnetic interaction between copper(II) ions in crystal lattice. Complexes were successfully evaluated for their cytotoxic activities on the normal human dermal fibroblast (NHDF) cell line and the antitumor activity using the human lung carcinoma (A549), epithelioid cervix carcinoma (HeLa), colon (LoVo), and breast adenocarcinoma (MCF-7) cell lines. Complexes **1** and **3** revealed lower toxicity to NHDF than A549 and HeLa cells, meanwhile compound **2** appeared to be more toxic to NHDF cell line in comparison to all cancer lines. Additionally, interactions between the complexes and human apo-transferrin (apo-Tf) using fluorescence and circular dichroism (CD) spectroscopy were also investigated. All compounds interacted with apo-transferrin, causing same changes of the protein conformation. Electrostatic interactions dominate in the **1**/**2** – apo- Tf systems and hydrophobic and ionic interactions in the case of **3**.

## 1. Introduction

Coordination chemistry of copper compounds has been an important subject of intensive investigations by researchers for many years [1,2,3]. Coordination polymers or discrete complexes constructed of copper metal ions and organic ligands/linkers have been investigated for the design of a new generation for luminescent [4,5,6,7], magnetic [8], catalytic [9,10,11,12], host-guests [13], and biological [14,15,16,17,18,19] properties. Thus, recently, there is great attention on the coordination chemistry of copper(I/II) complexes, due to their structural and physicochemical properties, in particular biological. On the one hand, an important role of copper in various biological processes, such as photosynthesis and dinitrogen metabolism as well as oxidative stress protection, etc., was reported by many scientists [14,15]. On the other hand, recent biological studies demonstrated that copper(I/II) coordination compounds represent various biomedical applications [16] that contain antifungal [17] and antibacterial [18] activities. Moreover, some of these complexes show also a relatively high activity as antiviral, anticancer, and antiproliferative agents and lower toxicity than cisplatin [15,17,18,19].

**Scheme 1:**
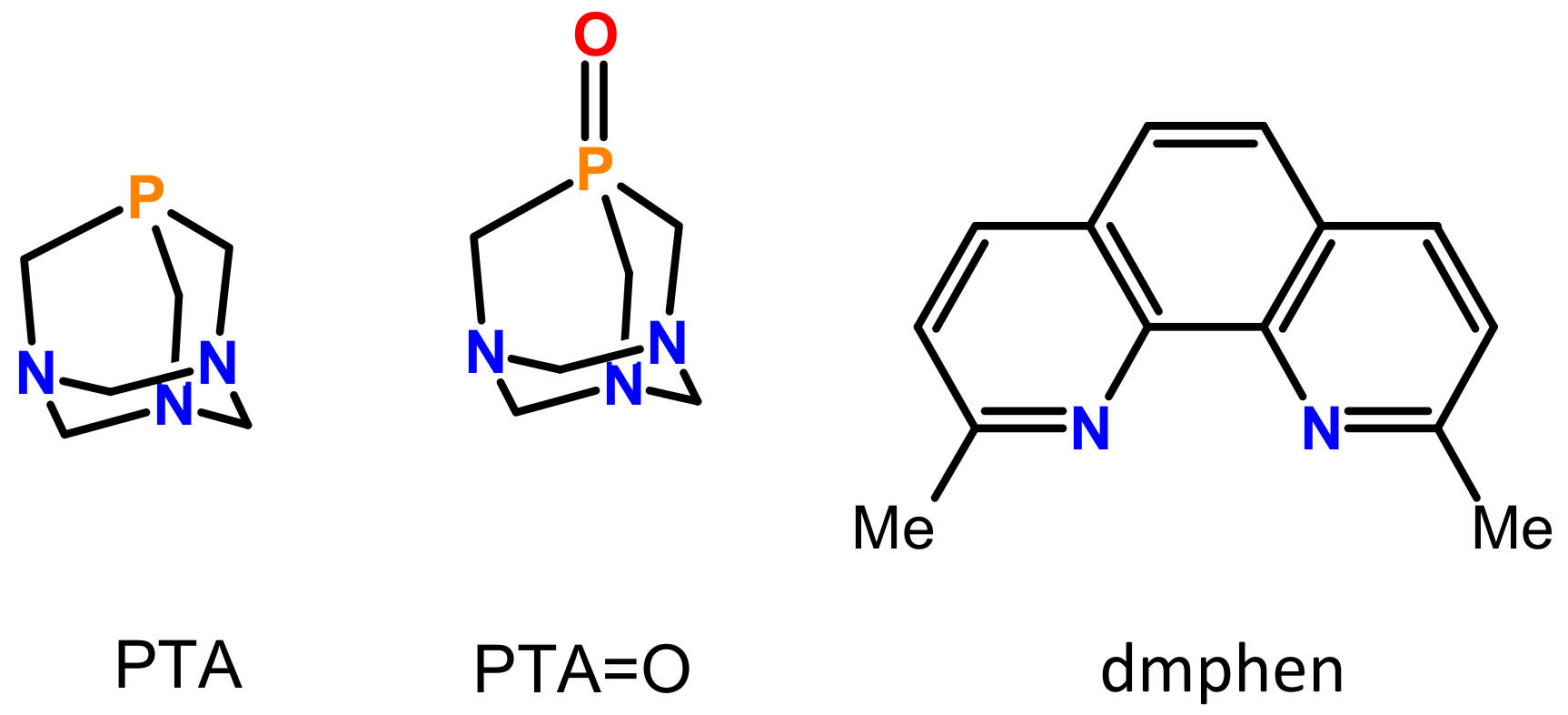
Structural formulas of the ligands.

However, copper(II) simple compounds have a relatively low human toxicity in comparison to other transition metal ions [20]. Toxicity of such complexes is connected with coordinated organic ligands, which are required properties for therapeutic agents. Additionally, many metal complexes used as bioactive agents are sparingly soluble in water media, limiting their applications in these systems. Combining of metal salts, organic ligands, and auxiliary counterions with appropriate properties may result not only in obtaining complexes with broader spectra of bioactivity but also sufficient aqua-solubility. In this regard, the air-stable and water-soluble aminophosphine 1,3,5-triaza-7-phosphaadamantane (PTA) and 1,3,5-triaza-7-phosphaadamantane-7-oxide (PTA=O) (Scheme 1) represent a good alternative to conventional phosphine and aminophosphine ligands. In the last decades the coordination chemistry of PTA and PTA=O has seen a pronounced development justified by the search for water-soluble and stable transition metal complexes [21,22,23]. Currently, several examples of PTA=O coordination modes are known, where a tetrapodal ligand is connected to a metal center in the O- [24,25,26], N- [24,27,28], ON- [24,25], ONN- [10,29], NNN- [30], or ONNN- modes [29,31], achieving either discrete metallic units or polymeric compounds [32]. 

Among the biorelevant N-donor ligands suitable to afford new bioactive metal complexes, polypyridines represent a good choice due to a recognizable influence on a large variety of biological functions [33,34,35,36,37]. In fact, the aromatic *N*,*N*-ligands such as 2,9-dimethyl-1,10-phenanthroline (dmphen, Scheme 1) have been a subject of intense research owing to their documented action in diverse biological systems [38]. A series of Cu(I)-dmphen complexes stabilized by tertiary phosphines and other Cu(II)-dmphen coordination compounds have been also reported, but their poor solubility in polar solvents eventually prevented the application of these compounds as bioactive materials [38,39]. In particular, some of us have reported a number of water-soluble copper(I/II)-PTA-cage coordination networks [10,40,41,42,43,44] as well as a discrete complexes [32,40,41,42,43,45,46,47,48], which were evaluated successfully for their magnetic [10,43,45], catalytic [10,44], and luminescent properties [40,42,44,48].

By further extending these studies from polypyridine-type *N*,*N*-ligand - 2,9-dimethyl-1,10-phenanthroline (dmphen), we now report the synthesis, characterization, and biological activity of the three discrete copper(II) complexes [Cu(NO_3_)(PTA=O)(dmphen)][PF_6_] (**1**), [Cu(Cl)(dmphen)_2_][PF_6_] (**2**), and (Cu(NO_3_)_2_(dmphen)) (**3**). Due to appropriate hydrosolubility, compounds were evaluated for their cytotoxic and antitumor activity in aqua media. In addition, the interactions between the complexes and human apo-transferrin were investigated by circular dichroism (CD) and fluorescence spectroscopy.

## 2. Results and Discussion 

### 2.1. Synthesis and Characterization

**Scheme 2:**
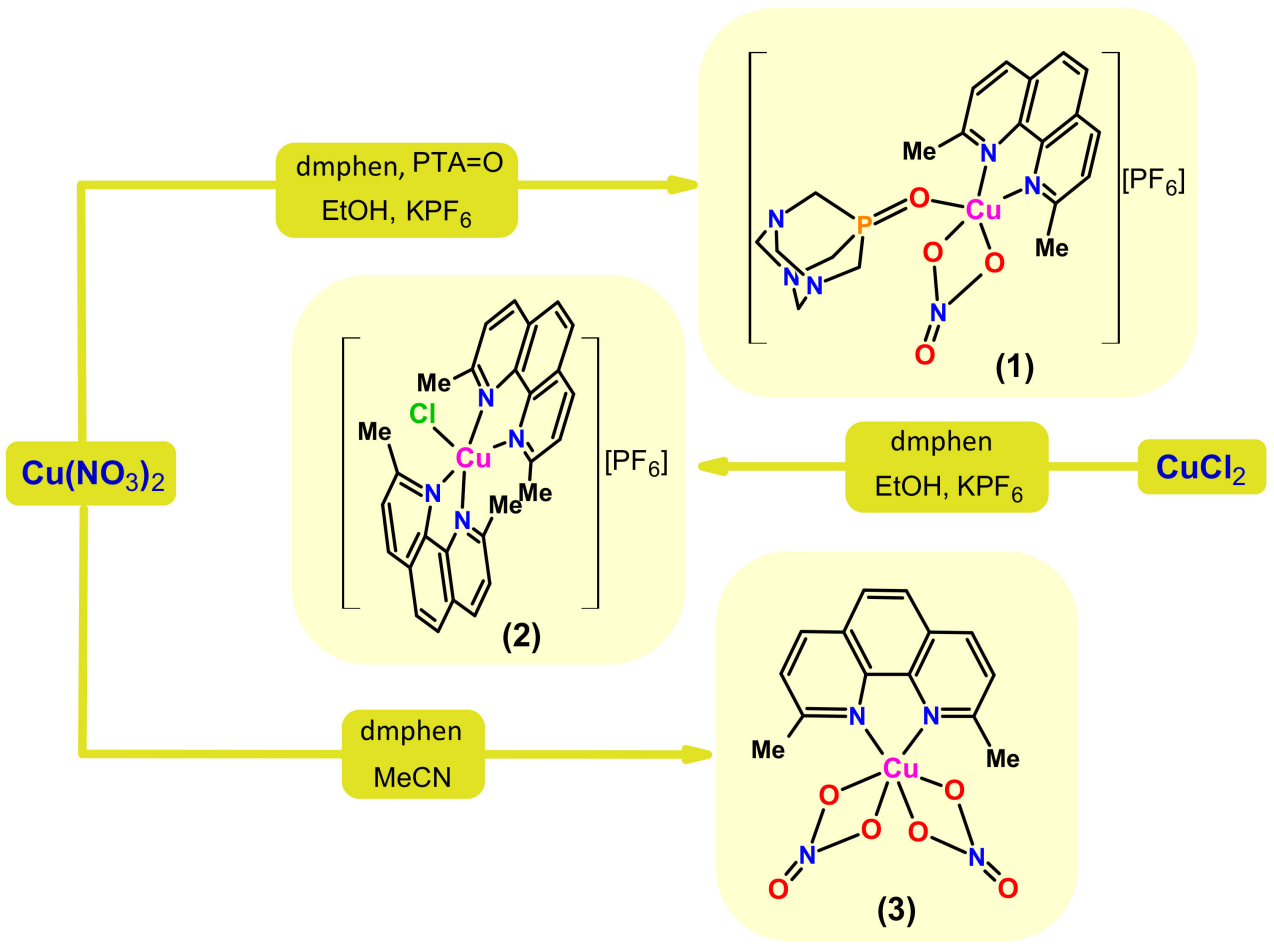
Schematic representation of the synthesis and structural formulae for **1**–**3**.

The reaction of Cu(NO_3_)_2_ with a stoichiometric amount of PTA=O, in EtOH solution under reflux conditions, followed by the addition of a stoichiometric amount of dmphen (Cu:PTA=O: dmphen molar ratios of 1:1:1), led to [Cu(NO_3_)(PTA=O)(dmphen)][PF_6_] (**1**) discrete coordination compound (Scheme 2). Treatment of CuCl_2_ with dmphen in the presence of KPF_6_ in a Cu:dmphen: KPF_6_ molar ratio (1:1:1) under the same conditions afforded [Cu(Cl)(dmphen)_2_][PF_6_] (**2**) complex (Scheme 2), whereas the use of Cu(NO_3_)_2_ salt with a stoichiometric amount of dmphen in MeCN under reflux conditions and molar ratio (1:1) gave rise to the formation of [Cu(NO_3_)_2_(dmphen)] ∙MeCN (**3**). Compounds **1**–**3** were isolated as air stable, green microcrystalline solids in ca. 36–85% yields based on appropriate copper salt, and characterized by IR spectroscopy, elemental analyses, and single-crystal X-ray diffraction. Although during the preparation of this manuscript the X-ray structure of **2** was published but described by us as one-pot reaction that was faster and yielded a cleaner product than the previously published method [49].

Compounds **1**–**3** were soluble in organic polar solvents, such as DMSO or MeOH, and sparingly soluble in middle polar solvents, like CH_2_Cl_2,_ whereas they were insoluble in apolar solvents, like Et_2_O, toluene, and alkanes. Moreover, all compounds were soluble in water, with the S_25 °C_ values 3.8, 2.5, and 2.9 mg mL^−1^ for **1**, **2,** and **3**, respectively. Their water solutions were relatively stable in air (see experimental section). The IR spectra of **1**–**3** exhibited absorptions due to the typical vibrations of dmphen, and, additionally, for **1**, PTA=O ligands with characteristic band at 1158 cm^−1^ due to ν(P=O) vibrations [21,22,23,50,51,52,53]. Additionally, the IR spectra also showed the characteristic strong and broad absorptions for NO_3_^−^ (for **1** and **3**), and PF_6_^−^ anions (for **1** and **2**), centered at 1365–1445 and 732–792 cm^−1^, respectively [53].

### 2.2. Crystal Structures

**Figure 1:**
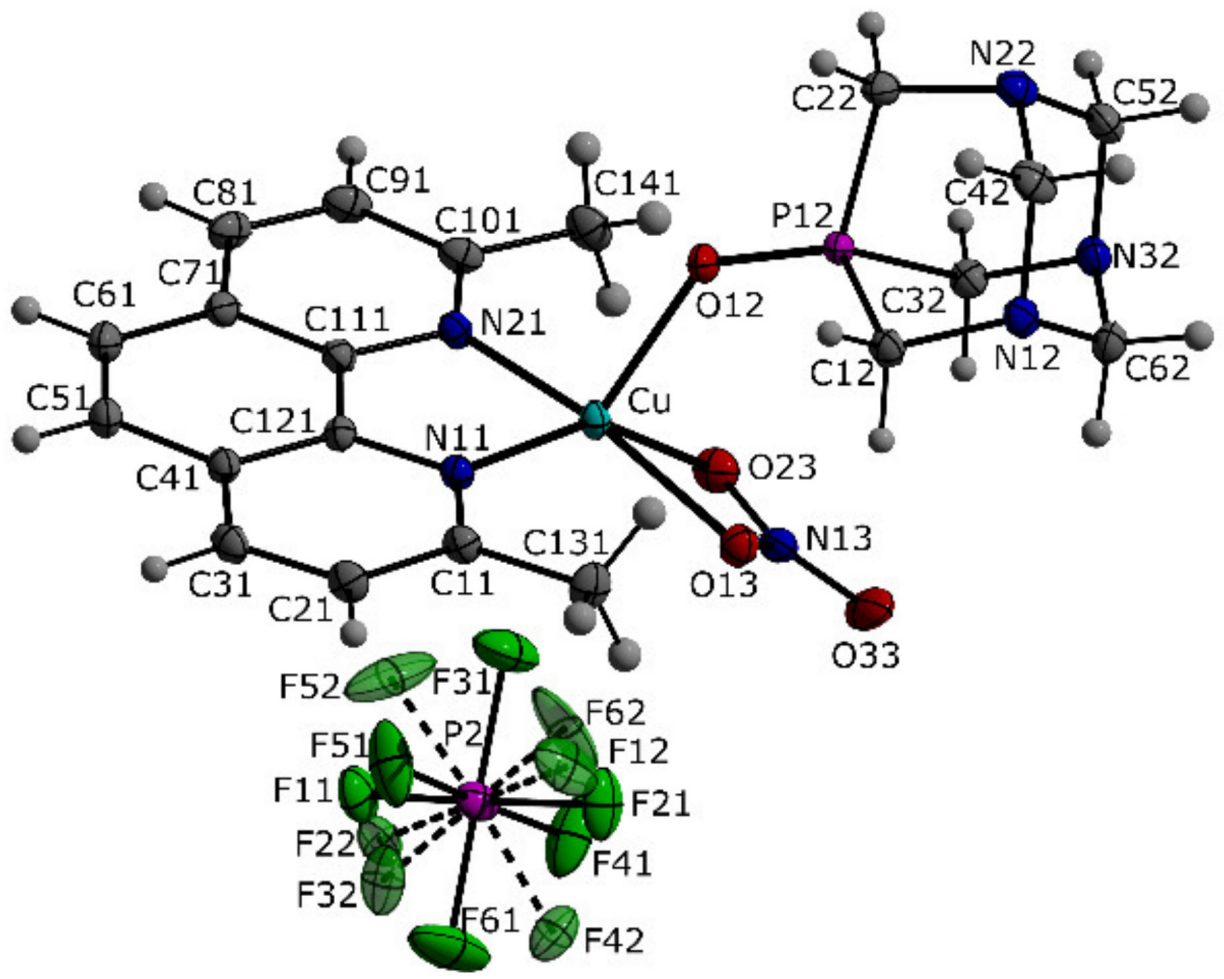
Molecular structure of compound **1**. Ellipsoids shown at the 50% probability. Color code: Grey, carbon; blue, nitrogen; red, oxygen; violet, phosphor; green, fluoride; cyan, copper; light grey, hydrogen. Selected bond lengths [Å] and angles [°]: Cu-N21, 1.972(1); Cu-N11, 1.995(1); Cu-O12, 2.102(1); Cu-O13, 2.028(1); Cu-O23, 2.083(1); P12-O12,1.506(1); N11-Cu-N21, 84.66(5); Cu-N11-C11, 130.80(9); Cu-O12-P12, 134.23(7); N11-Cu-O12, 112.83(4); N11-Cu-O13, 102.90(5); N11-Cu-O23, 148.04(5); N11-Cu-O12, 100.93(5); N21-Cu-O13, 161.17(5); N21-Cu-O23, 101.54(5); N21-Cu-O13, 92.08(4); O12-Cu-O23, 96.90(5); O13-Cu-O23, 63.04(5).

Compound **1** crystallized in monoclinic space group *P*2_1_*/n* and was composed of a [Cu(PTA=O)(dmphen)(NO_3_)]^+^ unit and PF_6_^−^ anion (Figure 1, Table 1). Compound **1** represented the first example of a Cu-dmphen five-coordinate complex with unusual bidentate coordination of NO_3_^−^ anion. The coordination geometry of Cu can best be described as distorted square–pyramidal with τ = 0.17 (where τ is the Addison parameter describing the distortion around coordination geometry, defined as τ  =  (β − α)/60 for α(O13–Cu–N11) = 102.90(4) Å and β(O12–Cu–N11) = 112.83(4) Å) [54]. The coordination sites of Cu were occupied by two nitrogen atoms from dmphen (N11 and N21), one oxygen atom from PTA=O (O12), and two oxygen atoms from NO_3_^−^ (O13 and O23). PF_6_^−^ anion was disordered over two positions. The Cu—N bonds were 1.995(1) Å (Cu—N11) and 1.972(1) Å (Cu—N21) and were a similar to those observed in other Cu—N-dmphen five-coordinate compounds (in a range of 1.988–2.039 Å), for example, (Cu(dmphen)(CH_3_OH))_2_(*µ*-C_2_O_4_))(ClO_4_)_2_ [55] and (CuCl_2_(dmphen)(H_2_O)) [56] complexes with Cu—O distances 1.970 Å and 1.996 Å, respectively. The Cu—O_NO3_ bond distances were 2.028(1) Å (Cu—O13) and 2.083(1) Å(Cu—O23) and were lower in comparison to values observed in other Cu—NO_3_ compounds, for example (Cu(C_7_H_5_O_3_)(NO_3_)(C_14_H_12_N_2_)) compound with Cu – O distances 2.292(3) Å [57].

**Figure 2:**
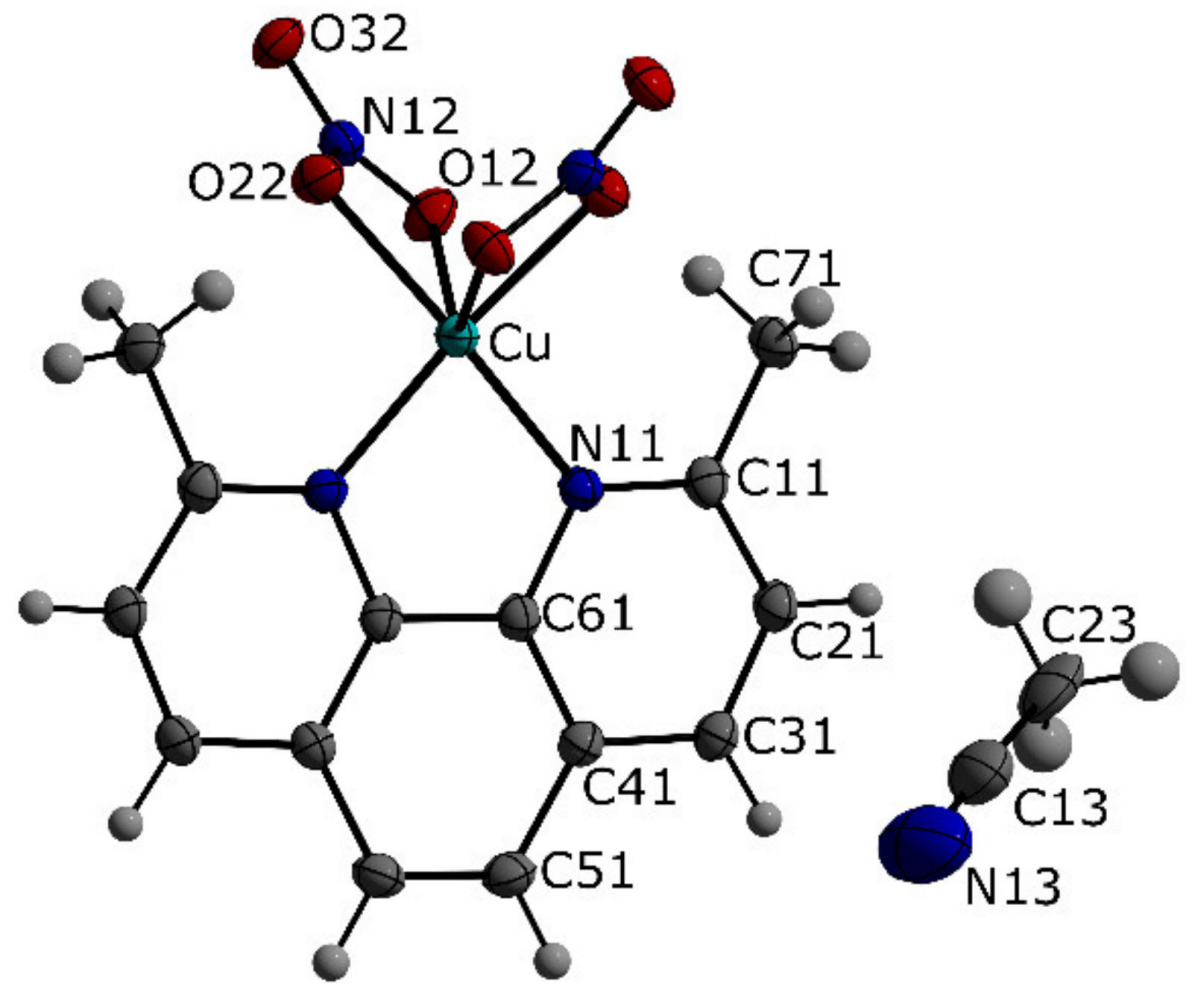
Molecular structure of compound **3** with atom labelling scheme. Ellipsoids shown at the 50% probability. Color code: Grey, carbon; blue, nitrogen; red, oxygen; cyan, copper; light grey, hydrogen. Selected bond lengths (Å) and angles (°): Cu-O12, 2.42; Cu-O22, 2.006; Cu-N11, 1.989; O12-Cu-O22, 57.62; O12-Cu-N11, 96.32; O12-Cu-N11, 119.68; O12-Cu-O12, 131.58; O12-Cu-O22, 87.13; O22-Cu-N11, 152.35; O22-Cu-N11, 99.67; O22-Cu-O22, 88.88; N11-Cu-N11, 84.89; N11-Cu-O22, 99.67.

The compound **3** crystallized in a monoclinic space group *I2/a*, comprising a Cu(dmphen)(NO_3_)_3_ unit with one solvent molecule (Figure 2, Table 1) and represented the second example of a Cu-dmphen six-coordinate complex with unusual bidentate coordination of NO_3_^−^ anion [58]. The coordination sphere of the Cu atom was occupied by two N atoms from dmphen and four O atoms from two nitrate anions, yielding a strongly distorted CuN_2_O_4_ octahedral environment. The Cu—N bond distance was 1.989(2) Å (Cu—N11) and was similar to those observed in other Cu—N-dmphen compounds [59]. The Cu—O bond distances were 2.421(2) Å (Cu—O12) and 2.006(2) Å (Cu—O22) and were comparable to Cu—O bond distances in Cu—NO_3_ compounds [60,61].

**Figure 3:**
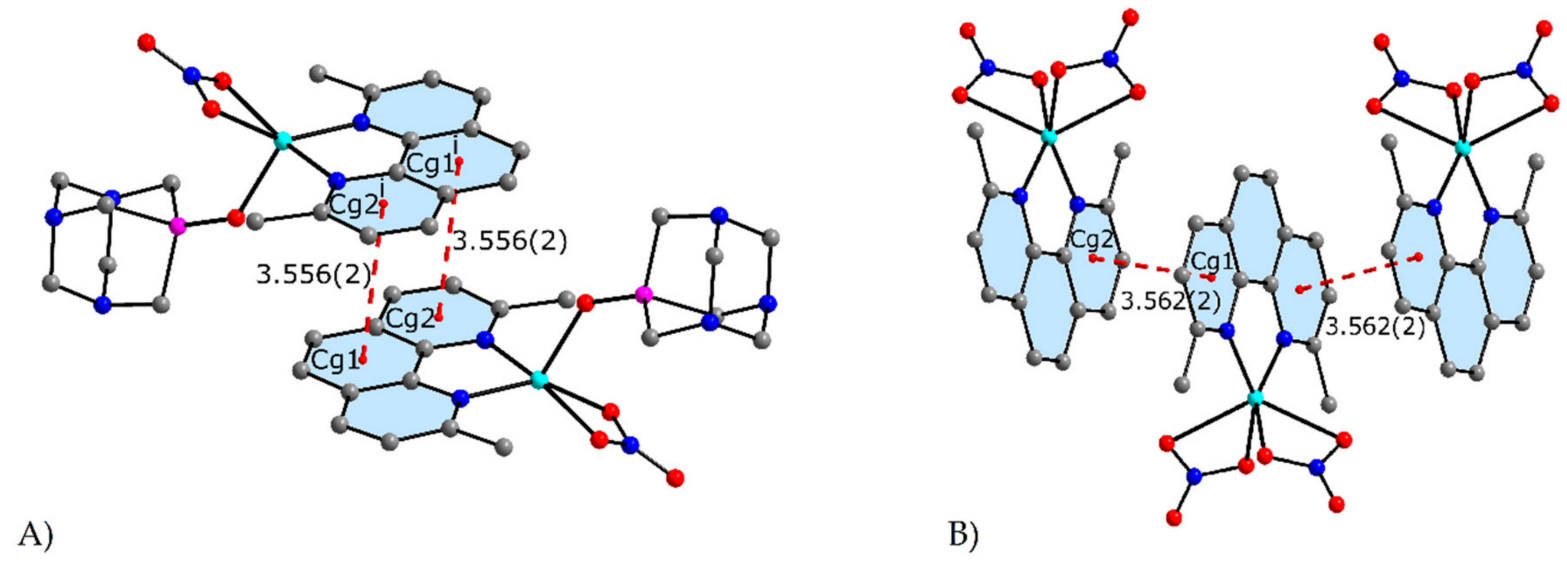
The π-π stacking interactions represented in red dashed line in compound **1** (**A**) and compound **3** (**B**).

The crystal structure of **1** was stabilized by π–π stacking interactions between antiparallel aromatic rings, with interplanar distances of 3.556 (2) Å (Figure 3A) [62]. In the crystal structure of **3**, molecules created 1D chains through π−π stacking interaction (3.562(2) Å) between dmphen ligands (Figure 3B) [62]. A channel between the chains was occupied by acetonitrile solvent molecules.

### 2.3. Magnetic Properties

**Figure 4:**
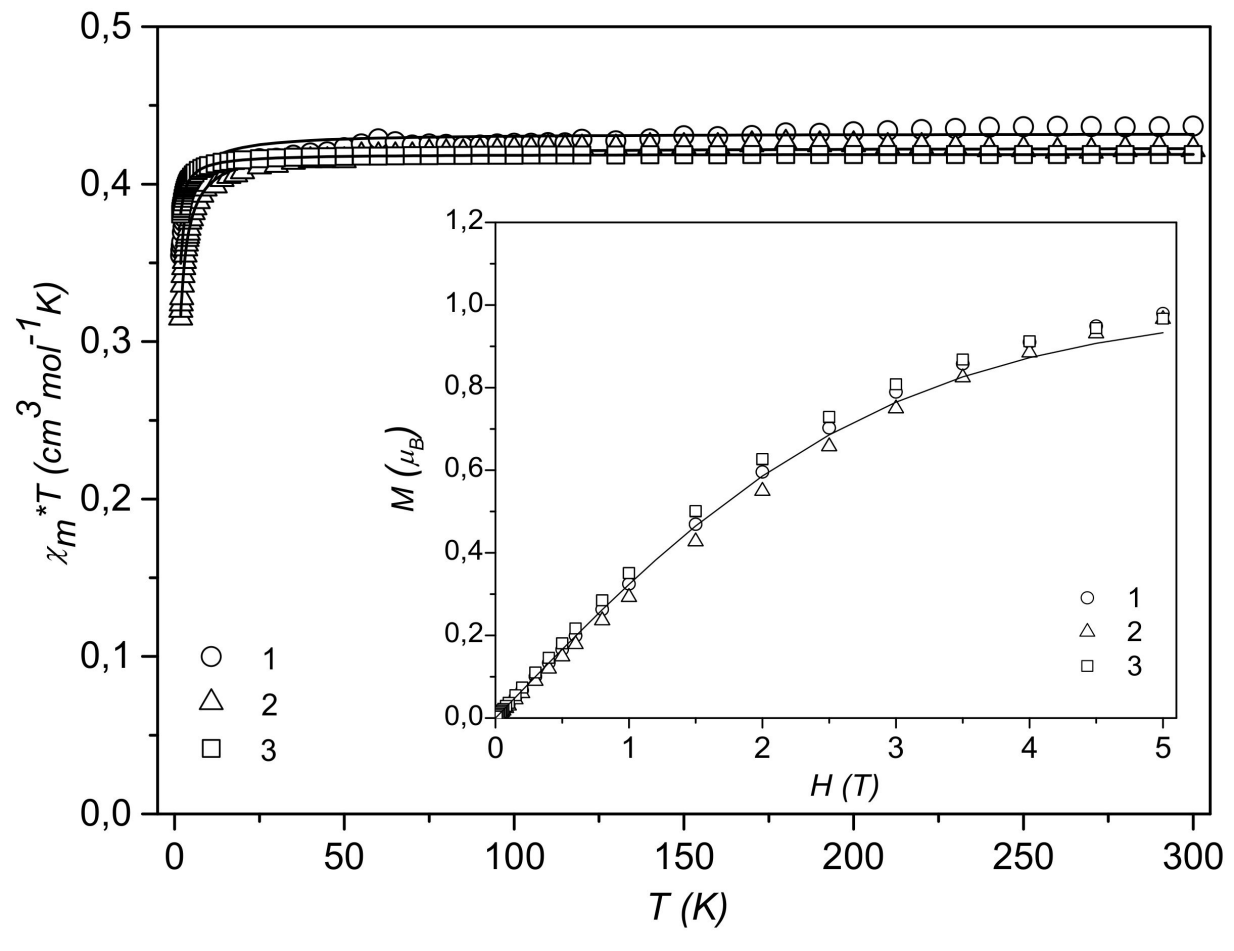
Temperature dependences of experimental *χ_m_T* vs. *T* (*χ*_m_ per one Cu^II^ atom) for **1–3.** The solid lines are the calculated curves derived from Equations (1) and (2). The inset shows field dependences of the magnetization (*M* per one Cu^II^ atom) at 2 K for **1–3**. The solid line is the Brillouin function curve for one uncoupled spin with *S* = ^1^/_2_ and *g* = 2.0.

The magnetic properties of **1**–**3** were investigated over the temperature range of 1.8–300 K. Plots of magnetic susceptibility *χ_m_T* product vs. *T* (*χ_m_* is the molar magnetic susceptibility per one Cu(II) ion) are given in Figure 4. For **1**–**3**, *χ_m_T* was essentially constant (~0.4 cm^3^ mol^−1^ K) in the whole temperature range. It was consistent with one unpaired electron in magnetically diluted copper(II) complexes [63]. Only a slight decrease of the value χ_m_*T* in the low-temperature range (below 10 K) was caused by occurrence of weak antiferromagnetic interactions in the crystal lattice. 

The variation of the magnetization (*M*) with respect to the field (*H*), at 2 K, also confirmed the nature of the ground state in **1**–**3** (Figure 4, see inset). As the magnetic field increased, the *M* vs. *H* curves are linear in the whole field range and indicate values of magnetization close to 1 *µ*_B_ at 5 T. The magnetization curves for **1**–**3** were reproduced by the equation $M=g\beta SNB_{s}(x)$ (S = 1·S_Cu_), where B_s_(x) is the Brillouin function and x = gβH/kT [63]. The experimental values closely followed the Brillouin function for one uncoupled spin with *S* = ^1^/_2_ and confirmed our previous assumption.

From the magnetic point of view, **1, 2,** and **3** were considered as mononuclear compounds. In such situations the magnetic data were fitted using the susceptibility equation for S = ^1^/_2_ (Equation (1)). To elucidate the significance of exchange between copper(II) ions in the crystal lattice, a molecular field correction term was also included (Equations (1) and (2)) [63,64].

(1) $$\chi_{M}=\frac{N\beta^{2}g^{2}}{3kT}S(S+1)$$

(2) $$\chi_{M}^{corr}=\frac{\chi_{M}}{1-\frac{2zJ^{’}}{N\beta^{2}g^{2}}\cdot \chi_{M}}$$

where *zJ*^’^ is the intermolecular exchange parameter, *z* is the number of the nearest neighbors, and the others have their usual meaning. A least-squares fitting of the experimental data led to the following values: *zJ’* = − 0.51(1) cm^−1^ and g = 2.10(1) (R = 3.47 × 10^−5^) for **1**, *zJ’* = − 0.79(1) cm^−1^ and g = 2.12(1) (R = 2.52 × 10^−5^) for **2,** and *zJ’* = − 0.45(1) cm^−1^ and g = 2.15(1) (R = 6.15 × 10^−5^) for **3,** as indicated by the solid curves in Figure 4. The calculated curves reproduced the magnetic data very well in the whole temperature range (Figure 4). The criterion used in determination of the best fit was based on minimization of the sum of squares of the deviation: *R =* Σ(*χ*_exp_*T* – *χ*_calc_*T*)^2^/Σ(*χ*_exp_*T*)^2^. Small *zJ’* exchange parameters (below 1 cm^−1^) were consistent with the crystal structures of **1–3**. Since the intermolecular Cu···Cu distances in all complexes were all rather long, it was to be expected that the coupling between electrons of the copper(II) ions in the system were weak. This fact was expected in mononuclear magnetically diluted Cu(II) compounds [65,66,67,68].

The EPR spectra of solid samples **1, 2,** and **3** recorded in the X-band at room temperature and 77 K were essentially similar and additionally confirmed the properties detected by the direct magnetic measurements. The EPR spectra of **1**–**3** resembled monomeric copper(II) species with poorly resolved hyperfine features and g_||_ > g_┴_ > g_e_. The spectral features were characteristic of a distorted square-planar geometry and a d_x_^2_^_y_^2^ ground state for the copper(II) center [69,70,71,72,73]. It was in fairly good agreement with the copper(II) geometries obtained from the crystal structures.

### 2.4. Cytotoxic Assays

On the one hand, toxicity levels of **1–3** on NHDF cells were investigated, which reflected normal cells of a body, and on the other hand, the ability of these compounds to kill the human lung (A549), breast (MCF7), colon (LoVo), and cervical (HeLa) cancer cells. These results of in vitro cytotoxicity tests are demonstrated in Table 2.

Complexes **1** and **3** revealed lower toxicity to normal human cell line (0.57 and 1.72 μM) than A549 cell (0.29 and 0.43 μM), respectively. Moreover, **3** showed similar activity against HeLa cell line (0.43 μM). In contrast to **1**–**3**, starting copper salt and free ligands in similar conditions had relatively less activity towards normal and cancer lines. Cisplatin displayed with regard to normal human cells weaker activity against cancer cell lines A549 and MCF7 and comparable against HeLa cell line. Similarly, complex **2** seemed to be more toxic in the case of NHDF cell line in comparison to all cancer lines.

It may be interesting to compare the activity of compounds **1**–**3** with the copper(I)-dmphen-phosphine complexes described in literature, especially as the influence of copper(I) compounds on cancer cells is well known. They usually show strong or moderate anticancer effect. However, they are not always safe for normal cells. According to Komarnicka et al. reported data, IC_50_ value for [Cu(iodide)(dmphen)(P(*p-*OCH_3_-Ph)_2_CH_2_OH)] showed 62.01 μM for A549 cell line, while, at the same time, already 32–43 μM was toxic for normal cell lines like MRC5, HEK293T, or HaCat [38]. In our case, not only that complexes **1** and **3** were less toxic for NHDF (normal cells) than A549, but also smaller than in the case of [Cu(iodide)(dmphen)(P(*p-*OCH_3_-Ph)_2_CH_2_OH)] doses (0.29 and 0.43 μM, respectively) were cytotoxic for cancer cells. A similar situation was observed in regard to [Cu(iodide)(dmphen)(P(*p*-OCH_3_-Ph)_2_CH_2_-SarGly)], where IC_50_ value for A549 was >100 μM. On the other hand, in the case of MCF7 cell line, both mentioned-above copper-iodide complexes showed better anticancer properties than **1** and **3**, which confirmed that the same compounds may be toxic for one type of neoplasia and nontoxic for others [38].

Cu(II) compounds acted differently on cells. Some of them, e.g., Schiff base copper(II) complexes type [Cu_2_(sal-d,l-glu)_2_(isoquinoline)_2_]∙2EtOH, [Cu(sal-5-met-l-glu)(H_2_O)]∙H_2_O, [Cu(EtOH)_2_(imidazole)_4_], and [Cu_2_(sal-d,l-glu)_2_(imidazole)_2_], showed weak (IC_50_ value >100 μM) cytotoxic effect on A549 or HeLa lines [76]. Others, guanidine copper(II) complexes type (Cu(C_4_H_3_SCONC(NHR)NPh)_2_) (R = aliphatic or aromatic groups), were cytotoxic for MCF7 and A549, but in much higher doses (61.1–370 μM) than **1**–**3** [77]. Toxicity of the aforementioned compounds for normal cells was also unknown [77]. Ganeshpandian et al. [78] reported Cu(II) compounds bearing dmphen ligand type [Cu(l)(dmphen)][ClO_4_]_2_ (l = substituted derivatives of amines), for which influence on MCF7 was tested. Series of these complexes were cytotoxic for human breast cells, but these doses, although lower than cisplatin, were several times higher (between 16.7 μM and 22.1 μM) compared to **1**, **2,** and **3**. In light of these studies, compounds **1**, **3,** and even **2** seem promising as potential anticancer agents, especially because, in contrast to cited-above reports, complexes, **1**–**3** were tested only in aqua media.

The bioactivities of the compounds is often compared with values of their logarithm of 1–octanol/water partition coefficient (log(P), see Experimental). This method describes hydrophobic/hydrophilic properties of the compounds. For the accurate biological activity and bioavailability of potential drugs, a balanced solubility in both water and nonpolar compounds such as lipids is essential [79]. Indeed, the different activities of the copper complexes **1–3** could be related to their positive, low log(P) values (0.90–1.75), in contrast to cisplatin with strongly negative log(P) factor (−2.21) [80].

### 2.5. Apo-Transferrin Interactions

**Figure 5:**
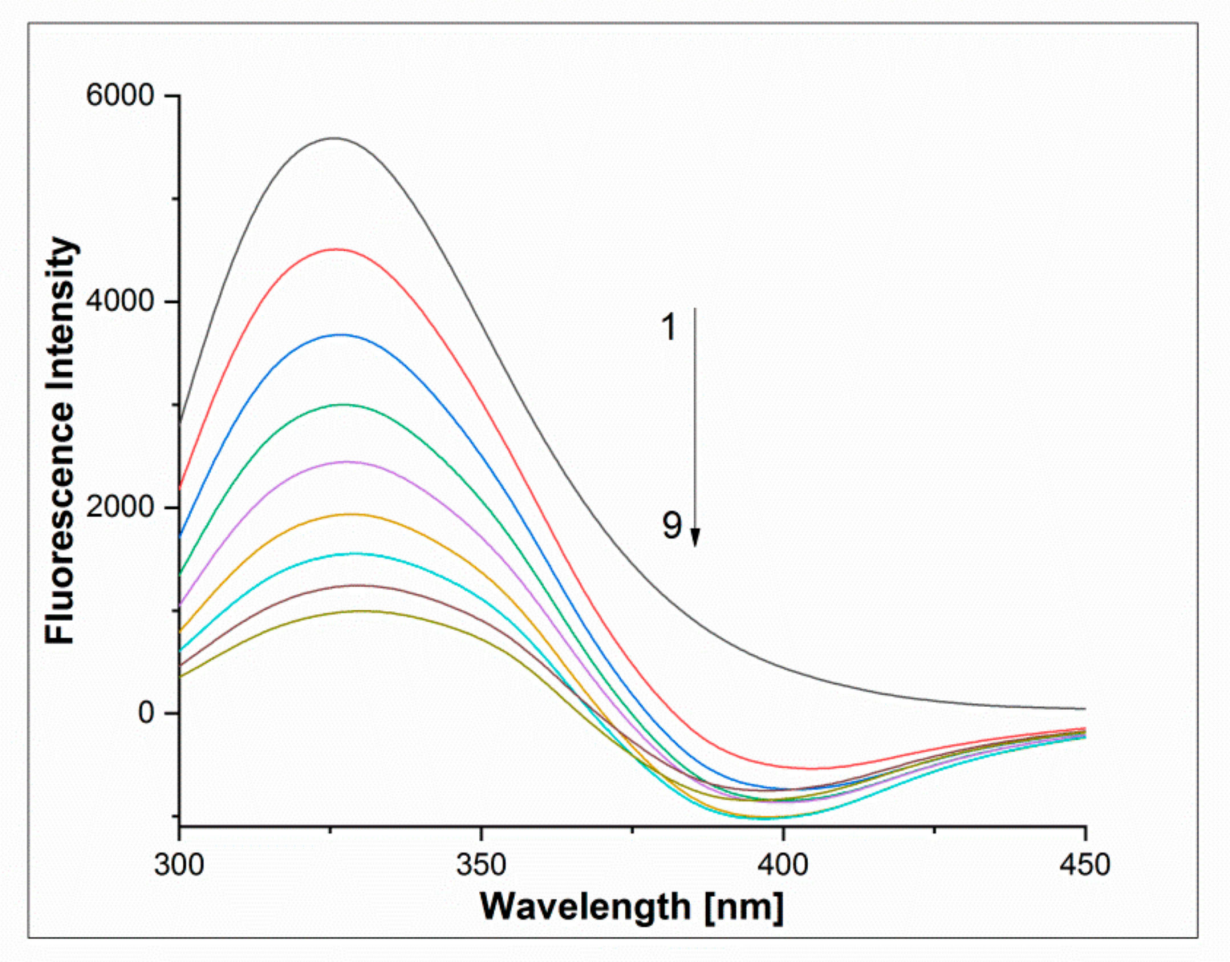
Fluorescence emission spectra of apo-Tf - **3** systems under physiological conditions (Phosphate-Buffered Saline-PBS, pH = 7.40, T = 310K), λ_ex_ = 280 nm, C_apo-Tf_ = 2·10^−6^M.

Figure 5 shows the fluorescence emission spectra of apo-Tf - copper complexes systems registered under physiological conditions and the excitation wavelength at 280 nm. It was evident that apo-Tf showed a strong fluorescence band at 325.5 nm and titration of the protein with small amounts of the complexes causing distinct decrease in fluorescence intensity of the protein. Moreover, the maximum band position was red shifted to ca. 330.5 nm. All the observations indicate that the apo-Tf conformation was changed and the protein’s chromophores were moved to a more polar environment [81].

There are two mechanisms of fluorescence quenching, dynamic and static, that can be distinguished based on the temperature dependency. Dynamic quenching is due to collisions between quencher and fluorophore and static quenching results from the formation of the ground state complex between the molecules [82]. A modified Stern–Volmer equation (Equation (3)) was used to calculate Stern–Volmer quenching constants (K_SV_) and quenching rate constants (Kq) of the interactions between apo-transferrin and copper complexes and to determine the mechanism of fluorescence quenching.

Fo/(Fo − F) = 1/fa + 1/(faKsv[Q])(3)

In the equation, Fo and F are the fluorescence intensities of protein in the absence and presence of the quencher in the concentration (Q), respectively; K_SV_ is the Stern–Volmer quenching constant; and fa is the fraction of the fluorophore accessible to the quencher.

Kq, the quenching rate constant of the biomolecules, is expressed as:

Kq = Ksv/τo(4)

where τo is the fluorescence lifetime of protein without quencher. For apo-transferrin, the τo value is 2.5 ns [83]. 

**Figure 6:**
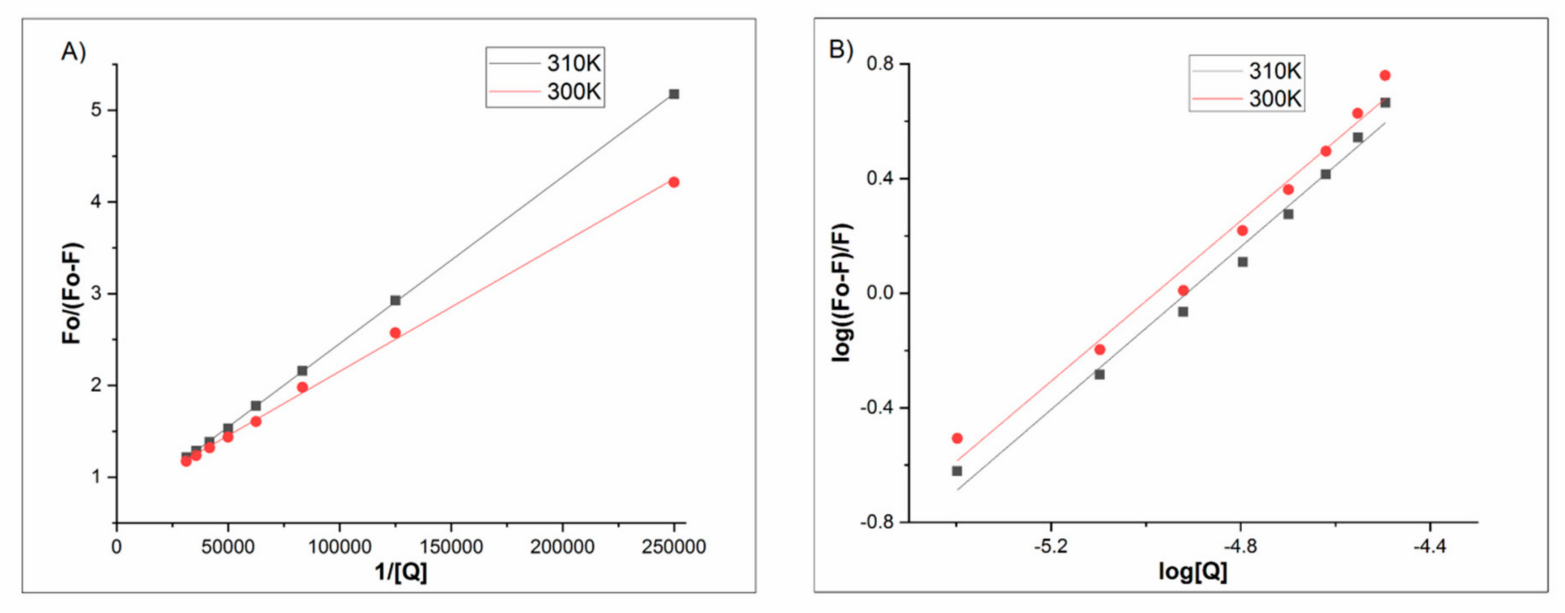
Modified Stern–Volmer (**A**) and log((F_0_ – F)/F) vs. log(Q) (**B**) plots of the apo-Tf- **3** system at 300 K and 310 K (PBS, pH = 7.40, λex = 280nm).

The modified Stern–Volmer plots are shown in Figure 6A, and Table 3 lists quenching parameters. As shown, the Ksv values increased with the temperature increases in the case of apo-Tf – complex **1** and complex **2** systems (2.50–5.69·10^4^ M^−1^). Simultaneously, the proper Kq values (10^13^ − 10^14^ (M^−1^s^−1^)) were higher than 2 × 10^10^ M^−1^s^−1^, the maximum Kq value expected for dynamic processes of the biopolymers [84,85]. Therefore, we concluded that some specific interactions were involved in the reaction between apo-Tf- copper complexes **1**/**2** that mad Kq greater, and the fluorescence quenching was initiated by both static and dynamic processes. In the case of apo-Tf – complex **3** system, the Ksv values (5.37 × 10^4^ and 3.51 × 10^4^ in 300 K and 310 K, respectively) were reversely correlated with the temperature, indicating static fluorescence quenching mechanism.

Association constants (Ka) and number of binding sites (n) were determined based on the Equation (5):

log {(Fo − F)/F} = logKa + nlog[Q](5)

where Fo and F are the fluorescence intensities in the absence and presence of the quencher in the concentration (Q), respectively, Ka is the binding constant, and n is the number of binding sites.

All binding data are collected in Table 4 and appropriate plots are shown in Figure 6B. It is clear that under tested conditions only one binding site (n) in protein for all copper complexes existed. The association constants (Ka) decreased with the temperature increases for **1** and **2**, suggesting forming of the unstable complexes. The binding constant of apo-Tf – **3** system increased with temperature increase, indicating the formation of the stable adduct and endothermic process. 

The interactions of the tested compounds with apo-Tf were definitely stronger than that of cisplatin (10^6^–10^7^ M^−1^ vs. 0.20–0.35 M^−1^, respectively) [86,87]. Moreover, obtained association constants were higher than association constant of chromium(III)-phen – apo-Tf system (1.5 × 10^5^ M^−1^) [88] or those obtained for NAMI-A (– Imidazolium-trans-tetrachloro(dimethylsulfoxide)imidazoleruthenium(III) – name explained in [89]) and its reduced form (1.28 × 10^4^ M^−1^ and 1.36 × 10^4^ M^−1^, respectively) [89].

It is known that interactions between proteins and small molecules include hydrogen bonds, van der Waals, and electrostatic forces as well hydrophobic interactions [90]. Based on thermodynamic parameters, we determined the type of interaction of copper complexes with apo-transferrin. Table 5 shows all ΔH^0^, ΔS^0^, and ΔG^0^ values obtained from van’t Hoff plots and Equations (6) and (7):

(6) $$\mathrm{lnKa}\;=\;-\;\frac{\Delta H^{0}}{RT}\;+\;\frac{\Delta S^{0}}{R}$$

∆G^0^ = ∆H^0^ − T∆S^0^ (7)

where Ka is the bimolecular binding constant at the corresponding temperature (T); R is the gas constant; and ΔH^0^, ΔS^0^, and ΔG^0^ are enthalpy, entropy, and free energy change, respectively.

The positive ΔS^0^ and negative ΔH^0^ for complexes **1** and **2** suggest that electrostatic interactions were involved in the reaction between protein and complexes. Both positive ΔH^0^ and ΔS^0^ for **3** indicate hydrophobic and ionic interactions. Negative values for all systems point to spontaneous processes.

**Figure 7:**
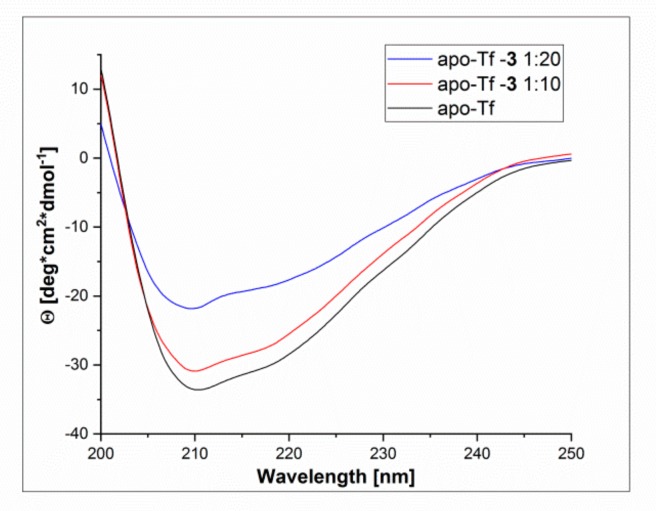
Circular dichroism -CD spectra of apo-Tf – **3** systems under physiological conditions (PBS, pH = 7.40, T = 310 K) (C_apo-Tf_ = 5 × 10^−6^ M).

Circular dichroism (CD) measurements in the far UV region were performed to determine the effect of copper complexes on the apo-transferrin secondary structure. A characteristic for α-helical structure of protein negative bands was visible in the CD spectrum at wavelengths of 210 and 220 nm (Figure 7) and were assigned *n-*π* transitions peptide bonds. All the tested complexes did not generate CD signal in the measured range. The α-helix content the free protein was equal to 19.80%. The interaction of **1** and **2** with apo-Tf had an insignificant effect on its secondary structure and, upon binding, the complexes α-helix content decreased to 19.07% and 18.00%, respectively, when the molar ratio apo-Tf:**1**/**2** was 1:20. In contrast to **1** and **2,** binding **3** complex to apo-transferrin caused extensive changes in conformation of the protein reducing α-helix content to 17.05% and 8.16% at the molar ratios apo-Tf:**3** equal 1:10 and 1:20, respectively.

## 3. Experimental

### 3.1. Materials and Methods 

All syntheses of compounds were carried out at room temperature (r.t.) in air. PTA=O (1,3,5-Triaza-7-phosphaadamantane-7-oxide) was synthesized following a reported procedure [91,92]. All other chemicals Cu(NO_3_)_2_∙3H_2_O (POCH, Gliwice, Poland), CuCl_2_∙2H_2_O (CHEMPUR, Piekary Śląskie, Poland), KPF_6_, dmphen (Sigma-Aldrich, St. Louis, USA), and analytical grade solvents, MeCN (Sigma-Aldrich, St. Louis, USA,), EtOH, and 1-octanol (POCH, Gliwice, Poland), were used without further purification. Elemental analyses were performed on the Elemental Analyser VarioELCube (Elementar Analysensysteme GmbH, Hanau, Germany) by the Laboratory of Elemental Analysis at Faculty of Chemistry, University of Wrocław. Infrared (IR) spectra were measured on a Bruker 70 Vertex 70 FTIR (Bruker, Ettlingen, Germany) instrument in the 4000–400 cm^−1^ range by the Laboratory of Infrared Spectroscopy at Faculty of Chemistry, University of Wrocław (abbreviations: vs, very strong; s, strong; m, medium; w, weak; vw, very weak; br., broad).

### 3.2. Cell Cultures

Normal human dermal fibroblasts (NHDF) (PromoCell, C-12302) were used as normal cells and LoVo (human colorectal adenocarcinoma, ATCC^®^ CCL-229™), A549 (human lung carcinoma, ATCC, No. CCL-185 TM), MCF-7 (human breast adenocarcinoma, ATCC, No. HTB-22^TM^) and HeLa (human cervix carcinoma, ATCC, No. CCL-2 TM) were used as cancer cells. They were cultured in DMEM, DMEM–F12 or EMEM (Lonza, CA, USA). Media were supplemented with 10% FBS (Biological Industries, Kibbutz Beit-Haemek, Israel), 2 mM L-glutamine (Biological Industries, Israel), 100 U/mL of penicillin and 100 µg/mL of streptomycin (Sigma, Neustadt an der Weinstrasse, Germany).

### 3.3. Cytotoxic Properties on Normal and Cancer Cell Lines - Quantitative Suspension Test According to EN 14476

The 100 μL of suspension of NHDF, A549, HeLa, LoVo, and MCF7 cells at a density of 4 × 10^4^ cells mL^−1^ were incubated in a 96-well polystyrene plate (NUNC, Denmark) for 24 h [93]. Product test solutions were prepared in DMEM, DMEM–F12, or EMEM supplemented with additional 2% FBS and 2 mM l-glutamine. Solutions of the reagents at concentrations from 460 (for **1**), 454 (**2**), 690 (**3**), 1242 (Cu(NO_3_)_2_), 1732 (PTA=O), and 1440 µM (dmphen) to 4.60 × 10^−6^, 4.54 × 10^−6^, 4.69 × 10^−6^, 1.24 × 10^−5^, 1.73 × 10^−5^, and 1.44 × 10^−5^ µM, respectively, were prepared and transferred (100 μL) into cell culture units (wells of microtiter plates) containing monolayer of cells. Eight units were inoculated with each dilution. Plates were incubated in 37 °C, 5% CO_2_ and observed daily for 4 days for the development of cytotoxic effect, using an inverted microscope (Olympus Corp., Hamburg, Germany, Axio Observer, Carl Zeiss MicroImaging GmbH). Then the wells were washed in phosphate buffered saline (PBS) and stained using DAPI (4′,6-diamidino-2-phenylindole) and propidium iodide (Merck, Darmstadt, Germany). The study used the fact that DAPI is able to be combined with cellular DNA, permeate through the membrane of cell, rapidly enter the nucleus of living cells and bind with DNA to form a DAPI-DNA complex. Propidium iodide does not pass through the membranes in healthy cells, but as a result of its damage it can penetrate into the cell. In cell culture these are cells that are apoptotic or damaged by any physical or chemical agent. Therefore, if these two fluorochrome are given at the same time, DAPI will get to healthy cells faster, while propidium iodide will stain dead or dying cells. The calculation of the ratio of cells counted on a specific area, allows to determine the potency of a substance in inhibiting a specific biological function (IC_50_).

### 3.4. Apo-Transferrin Interactions

High purity apo-transferrin (≥ 98%, Sigma-Aldrich, Steinheim, North Rhine-Westphalia, Germany) was used without prior purification. The stock solutions of the complexes were prepared in demineralized water (**1** and **2**) or ethanol (**3**). The apo-Tf was dissolved in PBS (pH 7.40). The molar ratios of the final samples in PBS were (protein):(drug) = 1:0–1:16 in PBS with the protein concentration equal 2 μM. Samples were incubated at 300 and 310 K for 5 min. The apo-Tf concentration was determined using ε (280 nm) = 11.2 mM^−1^ × cm^−1^.

#### 3.4.1. Fluorescence Spectroscopy

Emission fluorescence spectra were recorded on Jasco 8200 spectrofluorimeter (Tokyo, Japan) in the range of 300–500 nm using 1.0 cm quartz cells. The λex was set to 280 nm and the excitation and emission slit widths were set to 5 nm. All fluorescence intensities were corrected (according to Equation (8)) for the inner filter and dilution effects and the corrected values were used to determine the quenching mechanism and binding data. Moreover, the copper complexes showed a fluorescence signal in the measured range. Therefore, all spectra are shown as different spectra of (apo-Tf-copper complex)−(copper complex).

F_corr_ = exp(A_280_ + A_em_/2) × F_exp_ (8)

#### 3.4.2. CD Spectroscopy

Circular dichroism measurements were carried out on a Jasco J-715 spectropolarimeter (Tokyo, Japan) in the range of 190–250 nm using 0.1-cm cuvettes. The α-helical content of the protein was calculated from the Equations (9) and (10):

(9) $$MRE_{209}=\;\frac{Observed_{CD}\left(mdeg\right)}{C_{p}\cdot \;n\cdot \;l\;\cdot 10}$$

(10) $$\alpha -helix\;\left(\%\right)=\;-\frac{MRE_{209}-4000}{33000-4000}\times 100$$

where MRE is the mean residue ellipticity, *C_p_* is the molar concentration of the protein, *n* the number of amino acid residues (679), and *l* is the path length (0.1 cm).

### 3.5. Synthesis and Analytical Data

The [Cu(NO_3_)(PTA=O)(dmphen)][PF_6_] (**1**): 241.6 mg of Cu(NO_3_)∙3H_2_O (1.0 mmol) was dissolved in 40 mL of ethanol at room temperature and treated with 173.15 mg (1 mmol) solid PTA=O. During stirring for 1 h, the color of the solution changed from blue to green. Solid dmphen (208.25 mg, 1 mmol) with KPF_6_ (184.1 mg, 1 mmol) were added and the obtained mixture was refluxed for 2 h and then filtered off. The filtrate was left to slowly evaporate at ~4 °C for several days, producing green X-ray quality single crystals of **1** and red byproduct, determined as (Cu(dmphen)_2_) (PF_6_)_2_. The main product was washed with dimethylformamide/acetone and dried in air to furnish **1** in 36% yield (234 mg), based on the copper(II) salt. The **1** was soluble in H_2_O (S_25 °C_ ≈ 3.8 mg mL^−1^), DMSO, and MeOH; sparingly soluble in CH_2_Cl_2_ and EtOH; and insoluble in toluene and alkanes. Anal. Calcd. for C_20_H_24_CuN_6_O_4_P_2_F_6_ (MW 651.9): C, 36.85; H, 3.71; N, 12.89; found: C, 37,18; H, 3.62; N, 12.91. IR (KBr, cm^−1^): 3435 (s, br), 3065 (m), 2980 (m), 2923 (w), 2880 (m), 2546 (m), 2256 (m), 1996 (m), 1939 (m), 1837 (m), 1768 (m), 1710 (m), 1628 (m), 1618 (w), 1597 (s), 1572 (s), 1535 (vs), 1513 (vs), 1504 (vs), 1445 (s), 1430 (s), 1414 (s), 1384 (vs), 1365 (vs), 1303 (s), 1292 (vs), 1279 (vs), 1261 (vs), 1248 (vs), 1233 (vs), 1207 (m), 1158 (s), 1130 (vs), 1094 (s), 1044 (w), 1018 (s), 1006 (vs), 992 (m), 973 (vs), 943 (s), 910 (s), 869 (vs), 838 (vs), 814 (vs), 801 (vs), 792 (vs), 763 (s), 738 (m), 727 (s), 683 (m), 664 (w), 578 (s), 558 (vs), 470 (m), 450 (m), 442 (w), 432 (w), 421 (w), 388 (m).

The [Cu(Cl)(dmphen)_2_][PF_6_] **(2)**: The following one-pot reaction was faster and yielded a cleaner product than the previously published method [49]. The 170.5 mg of CuCl_2_∙2H_2_O (1.0 mmol) was dissolved in 40 mL of ethanol at room temperature and treated with 217.5 mg (1 mmol) dmphen and KPF_6_ (184.1 mg, 1 mmol). The obtained mixture was refluxed for 2 h and then filtered off. During the process the color of the cloudy solution changed from blue to yellow-green. The filtrate was left to slowly evaporate at ~4 °C for several days, producing green X-ray quality single crystals of **2** in 40% yield (265.7 mg), based on the copper(II) salt. The **2** was soluble in H_2_O (S_25 °C_ ≈ 2.5 mg mL^−1^), DMSO, and MeOH and EtOH; sparingly soluble in CH_2_Cl_2_; and insoluble in toluene and alkanes. Anal. Calcd. for C_28_H_24_ClCuN_4_PF_6_ (MW 660.5): C, 50.92; H, 8.48; N, 8.48; found: C, 50.90; H, 3.63; N, 8.44. IR (KBr, cm^−1^): 3272 (s), 3057 (w), 2923 (w), 1613 (m), 1593 (s), 1565 (m), 1505 (s), 1447 (m), 1426 (m), 1418 (m), 1364 (s), 1294 (w), 1251 (w), 1224 (w), 1215 (w), 1159 (w), 1147 (s), 1105 (w), 1035 (w), 996 (w), 987 (w), 940 (vw), 863 (vs), 841 (w), 813 (w), 801 (m), 776 (m), 732 (s), 697 (m), 682 (w), 656 (m), 581 (w), 550 (s), 434 (w).

The [Cu(NO_3_)_2_(dmphen)]∙MeCN **(3)**: 241.6 mg of Cu(NO_3_)∙3H_2_O (1.0 mmol) was dissolved in 50 mL of acetonitrile at room temperature and treated with 217.5 mg (1 mmol) dmphen. The obtained mixture was refluxed for 2 h and then filtered off. During the process the color of the solution changed from blue to green-brown. The filtrate was left to slowly evaporate at ~4 °C for one day, producing green X-ray quality single crystals of **3** in 85% yield (371 mg), based on the copper(II) salt. The **3** was soluble in H_2_O (S_25 °C_ ≈ 2.9 mg mL^−1^), DMSO, and acetone and MeOH; sparingly soluble in CH_2_Cl_2_; and insoluble in toluene and alkanes. Anal. Calcd. for C_16_H_15_CuN_5_O_6_ (MW 436.9): C, 43.99; H, 3.46; N, 16.03; found: C, 43.95; H, 3.43; N, 16.00. IR (KBr, cm^−1^): 3420 (s, br), 3073 (w), 2492 (m), 2285 (m), 2247 (m), 1991 (m), 1844 (m), 1767(m), 1719 (m), 1625 (m), 1594 (s), 1570 (s), 1500 (vs), 1365 (vs), 1273 (vs), 1248 (vs), 1217 (s), 1205 (s), 1156 (s), 1110 (m), 1034 (s), 1017 (vs) 1003 (vs), 979 (m), 943 (m), 865 (vs), 805 (s), 789 (m), 747 (m), 729 (s), 707 (w), 683 (m), 663 (m), 632 (w), 552 (m), 527 (m), 456 (m), 442 (w), 419 (w), 376 (vw).

### 3.6. Stability/Solubility Tests and Octanol-Water Partition Coefficient Determination

The copper complexes **1**–**3** were air stable in the solid state and at least for several days in H_2_O solutions. In a general procedure, the complex was dissolved in H_2_O in air atmosphere. IR spectra of the solid samples after evaporation of the solvent under vacuum showed that no evident changes were produced in several days at room temperature. Also UV-Vis spectra of water solutions of 1-3 confirm their stability in several days. In a stepwise procedure of the solubility determination, increasing volumes of water were added at 25 °C to approximately 5 mg of the compound in a 5 mL glass tube. After each addition of an amount of water (100 µL), the mixture was shaken for several minutes and then visually checked for any undissolved parts of the sample. 

The log P values consistent to the octanol-water partition coefficient were adjusted to the solubility properties of the compounds [94]. Complexes **1**–**3** were dissolved in water, previously saturated with octanol with concentrations 10^−5^ M. Into a 25-mL flask at room temperature with a magnetic stir bar was introduced initially 5 mL of octanol previously saturated with water and then 5 mL of the complex solutions in water. A two-phase mixture was stirred vigorously for 15 min and samples, which were measured by UV-Vis spectroscopy, were taken from the separated phases. Values of log P have been found as 0.90, 1.75, and 1.11, for **1**, **2,** and **3**, respectively.

### 3.7. X-ray Crystallography

Single crystal data collection was performed on KUMA diffractometer with Sapphire CCD detector, equipped with an Oxford Cryosystems open-flow nitrogen cryostat, using ω-scan and a graphite-monochromated Mo Kα (λ = 0.71073 Å) radiation. Cell refinement, data reduction, analysis, and absorption correction were carried out with CRYSALISPro (Rigaku Oxford Diffraction, Wrocław, Poland) software. The structures were solved by direct methods with SHELXS [95], and refined with full-matrix least-squares techniques on F^2^ with SHELXL [96]. The C-bonded hydrogen atoms were calculated in idealized geometry riding on their parent atoms. In the case of **1**, fluoride atoms were disordered over two equally occupied positions, while for **3**, acetonitrile molecule was disordered over two positions with occupation factor 0.5. As the crystals structure of compound **2** was already published (FEYTUB, CCDC 1819088) only preliminary data collection was performed to confirm identity [49].

The molecular structure plots were prepared using Diamond (Brandenburg, K. *Diamond*; Version 4.0; Crystal, Molecular Structure Visualization; Crystal Impact—K. Brandenburg and H. Putz Gbr: Bonn, Germany, 2009).

CCDC-1973604 (**1**) and CCDC-1973605 (**3**) contain the supplementary crystallographic data for this paper. These data can be obtained free of charge via http://www.ccdc.cam.ac.uk/conts/retrieving.html (or from the CCDC, 12 Union Road, Cambridge CB2 1EZ, UK; Fax: +44 1223 336033; E-mail: deposit@ccdc.cam.ac.uk).

### 3.8. Magnetic Measurement

The magnetization of powdered samples **1**, **2**, and **3** was measured over the temperature range 1.8–300 K using a Quantum Design SQUID-based MPMS-XL–5-type magnetometer (San Diego, USA). The superconducting magnet was generally operated at a field strength ranging from 0 to 5 T. Measurements were made at magnetic field 0.5 T. The SQUID magnetometer was calibrated with the palladium rod sample. Corrections were based on subtracting the sample-holder signal and the contribution of χ_D_ was estimated from the Pascal’s constants [97].

### 3.9. EPR Spectra

Electron Paramagnetic Resonance (EPR) spectra of powdered samples **1**, **2**, and **3** were recorded at room temperature and 77 K on a Bruker ELEXSYS E 500 CW-EPR (continuous-wave EPR) spectrometer (Billerica, MA, USA) operating at X-band frequency and equipped with an ER 036TM NMR Teslameter and E41 FC frequency counter.

## 4. Conclusions

A series of water-soluble and air-stable copper(II) discrete complexes based on 2,9-dimethyl-1,10-phenanthroline (dmphen) and mixed-ligands, containing PTA=O (1,3,5-triaza-7-phosphaadamantane-7-oxide), namely [Cu(NO_3_)(PTA=O)(dmphen)][PF_6_] (**1**), [Cu(Cl)(dmphen)_2_][PF_6_] (**2**), and [Cu(NO_3_)_2_(dmphen)] (**3**) were synthesized and fully characterized. The solid-state structures of all complexes were determined by single-crystal X-ray diffraction. Compound **1** extended a still poor family of Cu(II)-based metal-organic architectures assembled from a versatile, water-soluble, and cagelike aminophosphine oxide (PTA=O). The magnetic susceptibility measurements, as well as a relationship between the magnetization and magnetic field strength in **1**–**3,** revealed very weak antiferromagnetic interactions between magnetic centers of copper(II) ions in crystal lattice at low temperature.

Complexes were successfully evaluated for their cytotoxic activities on the normal human dermal fibroblast (NHDF) cell line and the antitumor activity using the human lung carcinoma (A549), epithelioid cervix carcinoma (HeLa), colon (LoVo), and breast adenocarcinoma (MCF-7) cell lines. Complexes **1** and **3** revealed lower toxicity (IC_50_) to normal human cell line (0.57 and 1.72 μM, respectively) than A549 cells (0.29 and 0.43 μM, respectively). Moreover, **3** showed similar activity against HeLa cell line (0.43 μM). Complex **2** was more toxic to NHDF cell line in comparison to all cancer lines. These results revealed anticancer potential of new compounds **1** and **3** and can be a starting point for further surveying of them as a chemotherapeutic agents in cancer treatment. In addition, all tested compounds interacted with human apo-transferrin, causing a conformational change of the protein. Complex **3** showed the most extensive interaction with the loss of helical stability of the protein. The positive values of ∆S^0^ and negative ∆H^0^ for apo-Tf –**1**/**2** systems indicated electrostatic interactions, and both positive parameters for **3** revealed hydrophobic and ionic interactions. Moreover, all reactions between copper compounds and human apo-transferrin were spontaneous processes.

## Figures and Tables

**Table 1:** Crystal data and structure refinement details.

Formula Unit	[C_20_H_24_CuN_6_O_4_P][PF_6_] (1)	[C_14_H_12_CuN_4_O_6_][C_2_H_3_N] (3)
Formula weight	651.93	436.87
Crystal system	Monoclinic	Monoclinic
Space group	*P*2_1_*/n*	*I2/a*
*a* (Å)	10.094(3)	10.500(7)
*b* (Å)	17.125(4)	14.480(8)
*c* (Å)	14.330(4)	12.426(9)
*β* (°)	95.44(3)	106.71(5)
*Z*	4	4
Volume (Å^3^)	2465.9(12)	1809(2)
*T (K)*	100(2)	100(2)
*D*_c_ (g cm^−3^)	1.756	1.604
*μ* (mm^−1^)	1.103	1.253
θ_max_, θ_min_ (°)	36.95, 3.50	28.69, 3.58
Rfls. measure, independent	39426, 11361	6557, 2183
R_int_	0.0539	0.0574
R_1_^a^,wR_2_^b^ [*I* *>* 2*σ*(*I*)]	0.0442, 0.1195	0.0530, 0.1365
R_1_, wR_2_ (all data)	0.0526, 0.1268	0.0604, 0.1548

*^a^* R1 = Σ||*F*_o_| − |*F*_c_||/Σ|*F*_o_|. *^b^* wR2 = [Σ[*w*(*F*_o_^2^ − *F*_c_^2^)^2^]/Σ[*w*(*F*_o_^2^)^2^]]^1/2.^

**Table 2:** IC_50_ values (μM) of the tested complexes (**1–3**), Cu(NO_3_)_2_, free ligands, and cisplatin.

Cell Line	1	2	3	Cu(NO_3_)_2_	PTA=O	Dmphen	Cisplatin
**NHDF**	0.57 ± 0.08	0.23 ± 0.03	1.72 ± 0.25	310 ± 47	nd	nd	16.6 ± 2.1 [74]
**A549**	0.29 ± 0.01	0.28 ± 0.04	0.43 ± 0.06	155 ± 23	nd	nd	33.3 ± 4.2 [74]
**HeLa**	1.12 ± 0.16	1.13 ± 0.17	0.43 ± 0.06	19.1 ± 2.9	nd	720 ± 108	16.6 ± 3.1 [74]
**MCF7**	0.57 ± 0.08	0.57 ± 0.08	3.45 ± 0.51	155 ± 23	nd	nd	33.3 ± 4.2 [74]
**LoVo**	0.57 ± 0.08	1.13 ± 0.17	1.72 ± 0.25	38.8 ± 5.8	nd	360 ± 54	9.12 ± 0.005 [75]

IC_50_—half maximal inhibitory concentration; nd = not detectable.

**Table 3:** The quenching parameters of the apo-Tf – Cu systems.

Parameter	Complex	300K	310K
**Ksv·10^4^ [M^−1^]**	**1**	2.50 ± 0.011	2.92 ± 0.029
	**2**	4.03 ± 0.050	5.69 ± 0.024
	**3**	5.37 ± 0.023	3.51 ± 0.062
**Kq·10^13^ [M^−1^s^−1^]**	**1**	1.00 ± 0.011	1.17 ± 0.029
	**2**	1.61 ± 0.050	2.27 ± 0.024
	**3**	0.12 ± 0.023	1.40 ± 0.062

**Table 4:** The binding parameters of the apo-Tf-Cu systems.

Parameter	Complex	300K	310K
**Ka·10^6^ [M^−1^]**	**1**	1.03 ± 0.16	0.843 ± 0.17
	**2**	4.38 ± 0.24	3.04 ± 0.32
	**3**	9.26 ± 0.37	9.38 ± 0.33
**n**	**1**	1.26 ± 0.033	1.24 ± 0.036
	**2**	1.35 ± 0.050	1.31 ± 0.066
	**3**	1.40 ± 0.076	1.42 ± 0.068

**Table 5:** Thermodynamic parameters of the apo-Tf - Cu systems at 300 and 310 K.

T [K]	H^0^ [kJ mol^−1^]			S^0^ [J mol^−1^ K^−1^]			G^0^ [kJ mol^−1^]		
	**1**	**2**	**3**	**1**	**2**	**3**	**1**	**2**	**3**
300	−15.33	−28.24	962.34	63.99	33.02	136.57	−34.53	−38.15	−40.01
310							−35.17	−38.45	−41.37
